# The effects of parental adverse childhood experiences (ACEs) and childhood threat and deprivation on adolescent depression and anxiety: an analysis of the longitudinal study of Australian children

**DOI:** 10.1017/S2045796025100255

**Published:** 2025-10-06

**Authors:** Santosh Giri, Nancy Ross, Rachel Kornhaber, Kedir Y. Ahmed, Subash Thapa

**Affiliations:** 1 Rural Health Research Institute (RHRI), Charles Sturt University, Orange, NSW, Australia; 2 School of Social Work, Dalhousie University, Halifax, NS, Canada; 3 School of Nursing, Paramedicine and Healthcare Sciences, Charles Sturt University, Bathurst, NSW, Australia

**Keywords:** adolescents, adverse effects, depression, epidemiology, maltreatment

## Abstract

**Aims:**

Evidence on the effects of parental Adverse Childhood Experiences (ACEs) on adolescent mental health remains limited. This study investigates the associations between parental ACEs, children’s exposure to threat- and deprivation-related ACEs, and adolescent depression and anxiety using data from the Longitudinal Study of Australian Children.

**Methods:**

We conducted a secondary analysis of the Longitudinal Study of Australian Children (LSAC), a population-based longitudinal cohort study. Parental ACEs were retrospectively reported by caregivers. Children’s exposure to ACEs was assessed from ages 4–17 years and categorised as threat-related ACEs (e.g., bullying, hostile parenting, unsafe neighbourhoods, family violence) or deprivation-related ACEs (e.g., financial hardship, parental substance abuse, parental psychological distress, death of a family member, parental separation, parental legal problems). Depressive and anxiety symptoms were self-reported by adolescents at ages between 12 and 17 years. Modified Poisson regression models were used to examine the independent and combined associations of parental ACEs and children’s threat- and deprivation-related ACEs (assessed before ages 12, 14, and 16 years) with depression and anxiety outcomes, including tests for interaction effects.

**Results:**

The analysis included 3,956 children aged 12–13 years, 3,357 children aged 14–15 years, and 3,089 children aged 16–17 years. Males comprised 50.8–59.8% and females 40.2–49.2% across all ages. By the age of 17, 30.4% and 9.4% of the adolescents had depression and anxiety, respectively. Parental ACEs (≥2) were associated with increased depression risk at ages 12 to 13 years (RR = 1.42; 95% CI: 1.10–1.84) and at 16–17 years (RR = 1.19; 95% CI: 1.02–1.39). Exposure to ≥ 2 deprivation-related ACEs significantly increased the risk of depression across all ages, with relative risks ranging from 1.31 to 2.18. High threat-related ACEs (≥2) were associated with increased depression risk only at 12 to 13 years (RR = 2.01; 95% CI: 1.28–3.17). No significant interactions were observed.

**Conclusions:**

The findings reinforce the ACEs model by showing that, at the population level, early identification of children exposed to early life deprivations rooted in financial crisis or familial adversities, combined with targeted interventions for both children and parents and supportive social policies, can reduce long-term mental health risks.

## Introduction

Adverse Childhood Experiences (ACEs), which include various forms of childhood maltreatment such as abuse, neglect, and household dysfunctions, are now widely recognised as persistent and pervasive early-life stressors that can profoundly shape developmental trajectories (Felitti *et al.*, 1998; Finkelhor *et al.*, 2015). Exposure to ACEs has been consistently linked to poor mental health outcomes, including heightened risk for depression and anxiety across the life course (Hughes *et al.*, 2017). These mental disorders, among the leading contributors to the global disease burden, account for 418 million disability-adjusted life years (16% of the global DALYs) and an estimated economic loss of $5 trillion (Arias *et al.*, 2022; Collaborators 2022). In children and adolescents, such conditions disrupt emotional well-being, academic performance, and social functioning, with effects often persisting into adulthood, amplifying the demand for specialised care, placing substantial strain on healthcare systems and economies globally (Mulraney *et al.*, 2021).

Importantly, ACEs are not uncommon in Australia, where a significant proportion of children experience at least one form of adversity during their formative years. A recent nationally representative sample, which assessed five types of child maltreatment, found that 32% of Australians reported physical abuse, 28.5% sexual abuse, 30.9% emotional abuse, and 39.6% reported exposure to domestic violence before the age of 18 years (Mathews *et al.*, 2023). Among youth aged 16–24 years, those who had experienced child maltreatment were approximately three times more likely to develop anxiety and major depressive disorders (Scott *et al.*, 2023). Further evidence indicates that higher ACE scores are strongly associated with increased externalising and internalising behaviours compared with peers who had lower ACE exposure (Gautam *et al.*, 2024). Similarly, bullying victimisation and parental psychological distress have been identified as major contributors to elevated anxiety and depressive symptoms, accounting for 47% of anxiety symptoms and 21% of depressive symptoms at the population level (Sahle *et al.*, 2024).

Although there is an abundance of evidence on the effects of ACEs on adolescent mental disorders (Scully *et al.*, 2020), the knowledge predominantly comes from studies analysing cumulative risk exposure to ACEs (i.e., summing the score of ACEs), assigning equal weight to each ACE type (Bomysoad and Francis, 2020; Hughes *et al.*, 2017; Merrick *et al.*, 2017; Metzler *et al.*, 2017). However, some ACE types, such as physical and sexual abuse, may be more influential than others when it comes to adolescent mental disorders (Ettekal *et al.*, 2019). Moreover, previous studies are limited by their focus on individual types of ACEs, reliance on cross-sectional or retrospective designs, and the confounding influence of life events occurring after childhood (Haynes *et al.*, 2020; Letourneau *et al.*, 2019; Sahle *et al.*, 2024). Longitudinal studies are necessary to provide deeper insights into the chronicity of trauma exposure among children, as well as the cumulative and long-term effects on their mental health.

The Dimensional Model of Psychopathology (DMAP) conceptualises ACEs as two distinct dimensions: threat and deprivation, each exerting independent effects on neurodevelopment and psychopathology through distinct pathways (McLaughlin *et al.*, 2014; Sheridan and McLaughlin, 2014). Threat-related ACEs, such as exposure to abuse, bullying, or witnessing violence, are characterised by direct harm or the anticipation of harm, leading to heightened stress reactivity and dysregulated emotional processing. In contrast, deprivation-related ACEs, including neglect and household dysfunction, are associated with the absence of expected social and cognitive inputs, potentially disrupting neurodevelopmental pathways involved in learning, attachment and social functioning (Henry *et al.*, 2021). To our knowledge, this is the first application of the DMAP framework to Australian longitudinal data, enabling an examination of how these distinct dimensions of adversity operate across adolescence.

Additionally, the ACEs of varying intensities may contribute to adolescent mental health not only through direct exposure but may also be driven by parental childhood trauma, leading to intergenerational trauma (Narayan *et al.*, 2021). The effects of parental ACEs may manifest in various ways, affecting parental practices or emotional availability, as well as hindering their ability to provide a safe and nurturing environment for their children (Cooke *et al.*, 2019; Luo *et al.*, 2023). These intergenerational effects may perpetuate cycles of adversity, increasing the risk of mental health disorders across generations (Thapa *et al.*, 2024). Understanding these pathways is crucial, as it could inform targeted interventions that address both child and parental trauma, with the potential to break the cycle of trauma, leading to more effective prevention and treatment strategies for mental health disorders in future generations.

By integrating an intergenerational perspective, differentiating ACEs into threat and deprivation dimensions, and utilising a longitudinal dataset, this study aims to advance existing knowledge on the pathways linking adversity to adolescent mental health in Australia. Therefore, this study investigated the independent impact of parental ACEs, children’s threat- and deprivation-related ACEs on depression and anxiety among Australian children at different ages (12–13, 14–15 and 16–17 years). We hypothesised that both parental ACEs and children’s threat- and deprivation-related ACEs would be independently associated with higher levels of depression and anxiety symptoms across adolescence. Furthermore, we expected that the impact of parental ACEs on adolescent mental health would be partially moderated by the child’s own exposure to ACEs.

## Methods

### Study design and population

**Figure 1.**
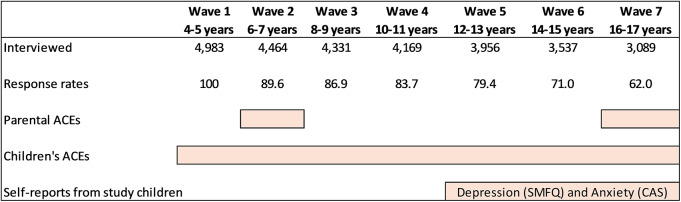
The longitudinal study of Australian Children: designs and measurement of key variables.

We conducted a secondary analysis of the Longitudinal Study of Australian Children (LSAC), a population-based longitudinal cohort study that employed a two-stage clustered sampling design, with recruitment beginning in 2004 (Figure 1). This study utilised the data from the kindergarten (K) cohort of LSAC, which consists of a sample of 4,983 Australian children. With biennial follow-up assessments, data collection was carried out through face-to-face interviews with multiple informants, including parents (mostly mothers or primary caretakers), children (when they reached the age of 12), teachers, and childcare workers. The detailed study design, sampling frame, sampling design and selection criteria have been published elsewhere (Soloff *et al.*, 2005).

We examined children’s ACEs reported by parent 1 for children aged 4 to 17 years (Waves 1–7), and parents’ ACEs reported by both parents at Waves 2 and 7. Depression and anxiety outcomes were self-reported by adolescents during Waves 5–7 when they were aged 12 years or older. The initial sample included 4,983 participants at Wave 1, with follow-up sizes of 3,956, 3,537, and 3,089 at Waves 5, 6, and 7, respectively. Seven data points in total were analysed over the 12-years follow-up period.

### Outcomes

Depression was measured using the Short Mood and Feelings Questionnaire (SMFQ), a 13-item validated self-reported measure of depressive symptoms administered for children and adolescents aged 8–16 years. For each item, the response options were true (coded as 2), sometimes true (coded as 1) and not true (coded as 0). The total score of the SMFQ scale ranges from 0 to 26. Using a cut-off score of ≥11, elevated depressive symptoms was defined as a dichotomous variable as having depression at 12–13, 14–15 and 16–17 years. A score of 11 or higher reliably identifies those meeting criteria for major depressive disorder (Turner *et al.*, 2014).

Anxiety was assessed using the Children’s Anxiety Scale (CAS-8), an 8-item self-reported measure of anxiety derived from Spence’s Anxiety Scale short form. These responses were recorded on a 4-point scale with options ranging from 0 to 3 (Never, Sometimes, Often and Always). Based on Spence *et al.* (2019), we defined anxiety symptoms using a total CAS-8 score of ≥13 for males and ≥ 16 for females, as these cut-offs were considered representative of clinical levels of anxiety symptoms, and were defined as a dichotomous outcome (Spence *et al.*, 2019).

The internal consistency of the SMFQ and CAS-8 scales across waves 5 to 7 was high, with Cronbach’s alpha coefficients ranging from 0.94 to 0.96 for depression and 0.89 to 0.93 for anxiety. Only a small proportion of children did not respond to the depression and anxiety questions at each wave (ranging from 2.8% to 5.3%). All other participants completed the full set of items for each scale, with no partial responses.

### Exposures

Children’s ACEs were reported by parent 1 and self-reported at multiple time points between ages 4 to 16 years. Based on the prior research identifying ACEs-related variables in the LSAC dataset (O’Connor *et al.*, 2020; Priest *et al.*, 2022; Sahle *et al.*, 2024), we defined ten types of adverse experiences that were consistently measured in the literature and had repeated measurements available from birth to adolescence. These measures included financial hardships, parental legal problems, interparental violence, parental psychological distress, having a household member with a drug or alcohol abuse problem, harsh parenting, parental separation or divorce, unsafe neighbourhood, death of a family member and bullying victimisation. Detailed definitions, operationalisation and timepoints of the adverse experiences used in this study are presented in Supplementary Table S1.

We created binary (yes/no) indicators for each ACE measure at each wave by dichotomising the respective items. To categorise the ACEs into threat and deprivation-related dimensions, four indicators of threat-related ACEs (family violence, hostile parenting, unsafe neighbourhoods, and bullying) and six indicators of deprivation-related ACEs (financial hardship, parental substance abuse, parental psychological distress, death of a family member, parental separation, and parental legal problems) were combined. We summed the number of ACEs related to threat and deprivation at each wave and grouped them into two categories: “≤1 ACEs” and “≥2 ACEs”, to reflect the potential impact of multiple adversities (Priest *et al.*, 2022). The final measure of threat and deprivation was a dichotomous value with 0 indicating “No” and 1 “Yes”, for exposure to high-level (≥2) threat- or deprivation-related ACEs. For the longitudinal analyses, threat- and deprivation-related ACEs were considered as exposures occurring before psychiatric outcomes, which were assessed at ages “12–13”, “14–15” and “16–17” years.

Parental ACEs were assessed through a series of questions administered to both parents across waves 2 and 7 (Supplementary file Table S2). These adverse experiences included authoritarian upbringing, lived in a foster family/welfare home/institution, family poor and struggled to meet ends, interparental violence (frequent arguments, abusive relationships and conflict), parent had mental illness, parent’s drinking/alcohol problem, neglected during childhood (frequently left alone not enough food in the house), received frequent beatings/physical punishment, sexually assaulted or abused and verbally abused. A cumulative parental ACEs score was derived, categorising parents based on whether they had experienced any childhood adversity. A dichotomous classification was used to define high exposure, with parents categorised as having experienced two or more ACEs. Details are presented in Supplementary Table S2.

### Covariates

Covariates were selected based on existing literature and included child’s sex (female/male), maternal age at birth (≥27 years/<27 years), family’s socioeconomic position (SEP) at Wave 1, and parental migrant status (at least one parent born overseas: no/yes). The cut-off for maternal age was defined as <27 years, representing the youngest quartile in the sample. SEP is a composite score of the z-score derived by standardising each parent’s annual income, education and occupation. For this study, SEP was categorised to quartiles, and a dichotomous variable was created, classifying families in the top 75% as “Advantaged” and the bottom 25% as “Disadvantaged”.

### Statistical analysis

The prevalence of depression and anxiety by high threat- and deprivation-related ACEs across the different stages of adolescence are reported as frequency and percentages with 95% confidence interval (CI). We used a modified Poisson regression model (Zou, 2004) with robust error variance to estimate the relative risk (RR) and 95% CI for the association between parental ACEs, children’s ACEs, and elevated symptoms of depression and anxiety at different stages of adolescence. This regression method has been widely used in prospective studies to estimate RR with a robust error variance as it provides a reliable way to estimate RRs directly, avoiding the biases of odds ratios from logistic regression and convergence issues of binomial models (Zou, 2004). To complement the modified Poisson regression, we replicated the analysis using the count outcomes of depression and anxiety and have presented both for comparison (Supplementary Tables S5 and S6).

Threat- and deprivation-related ACEs, assessed before ages 12, 14 and 16 years, were the primary exposures. Psychiatric outcomes, including depression and anxiety, were measured at ages 12–13, 14–15 and 16–17 years. The association between ≥2 threat- or deprivation-related ACEs before ages 12, 14, and 16 years, parental ACEs and elevated psychiatric symptoms at subsequent time points was examined. Analyses were conducted using unadjusted models and models adjusted for baseline confounders separately for depression and anxiety at each time point. Furthermore, we also conducted a full factorial three-way interaction between parental ACEs, and high threat- or deprivation-related ACEs at each wave. All analyses were weighted to account for the complex survey design and potential attrition bias. All statistical analyses were conducted using R Version 4.4.2 (R Core Team, 2024).

The percentage of complete cases ranged from 90% to 94.8% for both depression and anxiety at Waves 5 to 7 (with highest missing data on the parental ACEs). To address missing data in study variables, we performed multiple imputation by chained equations (MICE) using classification and regression trees separately for each wave (5, 6 and 7), following recommended practices (White *et al.*, 2011). The number of imputations was determined based on the extent of missingness within each wave, resulting in 50 imputed datasets per wave. The results from the multiple imputation were pooled using Rubin’s rule.

To assess the robustness of our findings from the multiple imputation (MI) dataset, we conducted two sensitivity analyses. First, we repeated all regression models using complete case data. Second, we re-estimated the models using continuous count scores of the outcome variables (rather than dichotomised cut-off values) on the imputed dataset.

### Role of the funding source

The funders had no role in study design, data collection, data analysis, interpretation or writing of the report.

### Ethics

The LSAC’s data collection protocols received ethical approval from the Australian Institute of Family Studies Ethics Committee. Written consent was obtained from parents, while children provided verbal assent.

## Results

### Study participants

The final analysis included 3,956 children aged 12–13 years in 2012, 3,357 children aged 14–15 years in 2014, and 3,089 children aged 16–17 years in 2016 (Table 1). Across all ages, the percentage of male participants ranged from 50.8% to 59.8%, while females comprised 40.2% to 49.2% of the sample for both threat- and deprivation-related ACEs. The majority of children had a primary caregiver aged ≥27 years at the child’s birth (over 70% for both exposures). Children from disadvantaged socioeconomic backgrounds consistently comprised over one-third of ACE-exposed cases—36.8% to 41.7%. The percentage of parents born outside Australia ranged from 48.0% to 56.2% for both exposures. Exposure to multiple parental ACEs (≥2) was common, affecting approximately two-thirds of parents, 62.1% to 69.8%.

**Table 1.** Sample characteristics of the study participants

	At 12−13 years	At 14−15 years	At 16−17 years
	(N = 3,956)	(N = 3,357)	(N = 3,089)
Characteristics	% (95% CI)	% (95% CI)	% (95% CI)
**Exposure to high threat-related ACEs**			
**Study child’s sex**			
Male	59.6 (55.1−63.9)	59.8 (55.5−64.1)	59.4 (54.7−63.8)
Female	40.4 (36.1−45.0)	40.2 (35.9−44.6)	40.6 (36.2−45.3)
**Age of the primary caregiver at birth of the study child**			
More than 27 years	72.5 (68.2−76.4)	73.8 (69.6−77.7)	73.4 (68.8−77.6)
Less than or equal to 27 years	27.6 (23.6−31.9)	26.2 (22.4−30.4)	26.6 (22.4−31.2)
**Family socioeconomic position at Wave 1**			
Advantaged (top 75%)	60.6 (56.0−65.1)	62.7 (58.2−67.0)	62.6 (57.7−67.2)
Disadvantaged (bottom 25%)	39.4 (34.9−44.0)	37.3 (33.0−41.8)	37.4 (32.8−42.3)
**Parent’s migrant status at Wave 1**			
Australian born	43.8 (39.5−48.3)	46.3 (42.0−50.7)	47.1 (42.4−51.7)
Non-Australian born	56.2 (51.7−60.6)	53.7 (49.3−58.0)	53.0 (48.3−57.6)
**Parental ACEs (≥2)**			
No/Limited (0 to 1 ACE)	35.7 (31.3−40.4)	37.9 (33.5−42.4)	34.0 (29.7−38.6)
Multiple (2 or more ACEs)	64.3 (59.6−68.7)	62.1 (57.6−66.5)	66.0 (61.4−70.3)
**Exposure to high deprivation-related ACEs**			
**Study child’s sex**			
Male	50.8 (47.1−54.6)	52.3 (48.7−56.0)	51.1 (47.3−54.9)
Female	49.2 (45.4−53.0)	47.7 (44.0−51.3)	48.9 (45.2−52.7)
**Age of the primary caregiver at birth of the study child**			
More than 27 years	72.0 (68.4−75.3)	72.8 (69.3−76.0)	73.3 (69.6−76.7)
Less than or equal to 27 years	28.0 (24.7−31.6)	27.2 (24.0−30.7)	26.7 (23.3−30.4)
**Family socioeconomic position at Wave 1**			
Advantaged (top 75%)	58.3 (54.5−62.1)	61.7 (58.0−65.3)	63.2 (59.3−67.0)
Disadvantaged (bottom 25%)	41.7 (37.9−45.5)	38.3 (34.7−42.1)	36.8 (33.0−40.7)
**Parent’s migrant status at Wave 1**			
Australian born	51.2 (47.4−54.9)	50.7 (47.1−54.4)	52.0 (48.2−55.8)
Non-Australian born	48.9 (45.1−52.6)	49.3 (45.6−52.9)	48.0 (44.3−51.8)
**Parental ACEs (≥2)**			
No/Limited (0 to 1 ACE)	31.9 (28.3−35.7)	34.7 (31.1−38.4)	30.2 (26.8−33.8)
Multiple (2 or more ACEs)	68.1 (64.3−71.7)	65.3 (61.6−68.9)	69.8 (66.3−73.2)

Data are % (95% CI) or *n*.

At ages 12–13 years, 14.5% (95% CI: 13.4–15.47) of children were exposed to high threat-related ACEs, and 20% (95% CI: 18.8–21.3) experienced high deprivation-related ACEs (Table 2). By ages 16–17 years, the exposure to high threat-related ACEs increased to 19.8% (95% CI: 18.4–21.3), and the percentage of children exposed to high deprivation-related ACEs reached 29% (95% CI: 27.5–30.7).

**Table 2.** Parental ACEs and high threat- and deprivation-related ACEs experienced between ages 4 and 17 years, anxiety and depression at ages 12–17 years

		High threat-related ACEs (≥2)		High deprivation-related ACEs (≥2)		Parental ACEs (≥2)	
	Total sample	Yes	No	Yes	No	No/limited (0 to 1 ACE)	Multiple (2 or more ACEs)
**At 12−13 years**							
Total	3,956	14.5 (13.4−15.7)	85.5 (84.3−86.6)	20 (18.8−21.3)	80 (78.7−81.2)	43.5 (41.9−45.1)	56.5 (54.9−58.1)
Elevated depression symptoms	11.2 (10.2−12.2)	16.2 (13.3−19.6)	10.2 (9.2−11.3)	18.7 (16−21.6)	9.3 (8.3−10.4)	9.3 (7.9−10.8)	12.4 (11−13.9)
Elevated anxiety symptoms	4.5 (3.9−5.2)	5.7 (4.0−7.9)	4.3 (3.6−5.0)	7.6 (5.9−9.6)	3.7 (3.1−4.4)	3.8 (3.0−4.9)	4.5 (3.7−5.5)
**At 14−15 years**							
Total	3,357	17.8 (16.5−19.1)	82.2 (80.9−83.5)	25 (23.6−26.4)	75.0 (73.6−76.4)	42.2 (40.6−43.9)	57.8 (56.1−59.4)
Elevated depression symptoms	19.0 (17.7−20.4)	23.3 (20.0−27.0)	18.4 (17.0−19.9)	26.5 (23.6−29.6)	16.6 (15.2−18.1)	17.8 (15.8−19.9)	19.6 (17.8−21.4)
Elevated anxiety symptoms	6.1 (5.3−6.9)	8.6 (6.6−11.1)	5.4 (4.7−6.4)	8.6 (6.9−10.6)	5.2 (4.4−6.1)	5.5 (4.4−6.8)	6.4 (5.4−7.6)
**At 16−17 years**							
Total	3,089	19.8 (18.4−21.3)	80.2 (78.7−81.6)	29.0 (27.5−30.7)	71.0 (69.3−72.5)	38.4 (36.7−40.1)	61.6 (59.9−63.3)
Elevated depression symptoms	30.4 (28.8−32.1)	34.9 (31.0−39.0)	29.4 (27.6−31.3)	36.6 (33.4−39.9)	27.9 (26.1−29.9)	27.7 (25.2−30.5)	32.1 (30−34.3)
Elevated anxiety symptoms	9.4 (8.4−10.5)	12.8 (10.4−15.8)	8.6 (7.5−9.8)	13.3 (11.2−15.6)	7.8 (6.7−9)	9.0 (7.5−10.8)	9.6 (8.4−11.1)

Data are % (95% CI) or *n*.

### Prevalence of depression and anxiety at different ages

At ages 12–13 years, the overall prevalence of depression was 11.2% (95% CI: 10.2–12.2) (Table 2). By ages 14–15 years, 19% (95% CI: 17.7–20.4) of the children reported depression, and at ages 16–17 years, it further increased to 30.4% (95% CI: 28.8–32.1). 16.2% (95% CI: 13.3–19.6) children exposed to high threat-related ACEs and 18.7% (95% CI: 16.0–21.6) children exposed to high deprivation-related ACEs at ages 12–13 years reported higher depression rates. By ages 16–17 years, the prevalence of depression was 34.9% (95% CI: 31.0–39.0) among those exposed to high threat-related ACEs and 36.6% (95% CI: 33.4–39.9) among those exposed to high deprivation-related ACEs.

Similarly, anxiety was reported among 4.5% (95% CI: 3.9–5.2) at 12–13 years and 9.4% (95% CI: 8.4–10.5) at 16–17 years. The children with high threat-related ACEs had a higher prevalence anxiety [5.7% (95% CI: 4.0–7.9) at 12–13 years vs. 12.8% (95% CI: 10.4–15.8) at 16–17 years], and those with high deprivation-related ACEs had a similar rate [7.6% (95% CI: 5.9–9.6) at 12–13 years vs. 13.3% (95% CI: 11.2–15.6) at 16–17 years].

Children born to parents with multiple parental ACEs (≥2) consistently reported higher rates of depression and anxiety as they aged. At ages 12–13 years, 12.4% (95% CI: (11.0–13.9) of these children had depression, which increased to 19.6% (95% CI: 17.8–21.4) at ages 14–15 years, and 32.1% (95% CI: 30.0–34.3) at ages 16–17 years. Similarly, the prevalence of anxiety also increased, from 4.5% (95% CI: 3.7–5.5) at 12–13 years to 6.4% (95% CI: 5.4–7.6) at ages 14–15 years, reaching 9.6% (95% CI: 8.4–11.1) at ages 16–17 years.

### Association between parental history of ACEs, child’s ACEs exposure and adolescent depression at different ages

The results from the final adjusted interaction model revealed that children exposed to ≥ 2 deprivation-related ACEs had a 2.18 times higher risk (RR = 2.18; 95% CI: 1.49–3.20) of depression at ages 12–13 years, 34% (RR = 1.34; 95% CI: 1.01–1.8) higher risk of depression at ages 14–15 years, and 31% (RR = 1.31; 95% CI: 1.04–1.66) higher risk of depression at ages 16–17 years compared to those exposed to lower deprivation-related ACEs at respective ages (Table 3). Threat-related ACEs showed a weaker association, with 2.01 times higher risk (RR = 2.01; 95% CI: 1.28–3.17) of depression at ages 12–13 years only. Similarly, children born to parents with multiple ACEs had 42% (RR = 1.42; 95% CI: 1.10–1.84) higher risk of depression at ages 12–13 years and 19% (RR = 1.19; 95% CI: 1.02–1.39) higher risk at ages 16–17 years. The three-way interaction was not significant at any time point.

**Table 3.** Modified Poisson regression model for the association between parental history of ACEs, child’s ACEs exposure (ages 3–16 years), and adolescent depression (ages 12–17 years)

	Depression at 12−13 years			Depression at 14−15 years			Depression at 16−17 years		
	Crude RR (95% CI)	Adjusted RR^a^ (95% CI)	Adjusted RR^a^ with interactions (95% CI)	Crude RR (95% CI)	Adjusted RR^a^ (95% CI)	Adjusted RR^a^ with interactions (95% CI)	Crude RR (95% CI)	Adjusted RR^a^ (95% CI)	Adjusted RR^a^ with interactions (95% CI)
**Study child’s sex**									
Male									
Female	1.13 (0.95−1.36)	1.15 (0.97−1.37)	1.16 (0.97−1.38)	1.89 (1.63−2.19)	1.92 (1.66−2.22)	1.92 (1.65−2.22)	1.48 (1.32−1.65)	1.48 (1.32−1.65)	1.48 (1.32−1.65)
**Age of the primary caregiver at birth of the study child**									
More than 27 years									
Less than or equal to 27 years	1.61 (1.32−1.97)	1.37 (1.11−1.68)	1.36 (1.11−1.68)	1.33 (1.12−1.57)	1.22 (1.03−1.45)	1.22 (1.03−1.45)	1.19 (1.04−1.37)	1.10 (0.95−1.26)	1.10 (0.95−1.26)
**Family socioeconomic position at Wave 1**									
Advantaged (Top 75%)									
Disadvantaged (Bottom 25%)	1.67 (1.38−2.02)	1.36 (1.11−1.66)	1.35 (1.11−1.65)	1.35 (1.15−1.58)	1.16 (0.99−1.37)	1.17 (0.99−1.37)	1.36 (1.20−1.55)	1.25 (1.10−1.42)	1.24 (1.09−1.42)
**Parents’ migrant status at Wave 1**									
Australian born									
Non-Australian born	0.98 (0.82−1.17)	0.98 (0.82−1.17)	0.98 (0.82−1.17)	0.98 (0.85−1.12)	0.97 (0.85−1.12)	0.97 (0.85−1.12)	0.9 (0.81−1.01)	0.90 (0.80−1.00)	0.90 (0.80−1.00)
**Parental ACEs (≥2)**									
No/Limited (0 to 1 ACE)									
Multiple (2 or more ACEs)	1.37 (1.13−1.66)	1.26 (1.04−1.53)	1.42 (1.10−1.84)	1.10 (0.95−1.27)	1.08 (0.93−1.24)	1.04 (0.86−1.26)	1.16 (1.03−1.30)	1.15 (1.02−1.29)	1.19 (1.02−1.39)
**Study child’s high threat related ACEs (≥2)**									
No									
Yes	1.61 (1.3−2.00)	1.29 (1.03−1.62)	2.01 (1.28−3.17)	1.26 (1.06−1.49)	1.16 (0.98−1.38)	1.09 (0.74−1.62)	1.18 (1.03−1.34)	1.10 (0.97−1.26)	1.27 (0.95−1.69)
**Study child’s high deprivation related ACEs (≥2)**									
No									
Yes	2.01 (1.67−2.41)	1.67 (1.37−2.04)	2.18 (1.49−3.20)	1.60 (1.39−1.85)	1.49 (1.28−1.73)	1.34 (1.01−1.80)	1.31 (1.17−1.47)	1.22 (1.08−1.37)	1.31 (1.04−1.66)
**Parental ACEs × High Threat ACEs × High Deprivation ACEs**									
Parental ACEs × High Threat ACEs	0.89 (0.46−1.71)	–	0.90 (0.47−1.72)	1.09 (0.63−1.91)	–	1.10 (0.63−1.90)	0.90 (0.60−1.37)	–	0.95 (0.63−1.44)
Parental ACEs × High Deprivation ACEs	1.06 (0.62−1.81)	–	1.09 (0.64−1.85)	1.16 (0.76−1.77)	–	1.20 (0.80−1.81)	1.06 (0.76−1.47)	–	1.07 (0.77−1.48)
High Threat ACEs × High Deprivation ACEs	0.61 (0.25−1.49)	–	0.64 (0.26−1.57)	1.33 (0.70−2.54)	–	1.28 (0.67−2.43)	0.92 (0.55−1.55)	–	0.90 (0.54−1.50)
Parental ACEs × High Threat ACEs × High Deprivation ACEs	2.92 (1.03−8.33)	–	2.82 (0.99−8.03)	0.76 (0.35−1.68)	–	0.83 (0.38−1.81)	1.84 (0.99−3.40)	–	1.80 (0.98−3.31)

a Adjusted for study child’s sex, age of the primary caregiver at birth of the study child, family socioeconomic position at Wave 1, Parents’ migrant status and Parental ACEs; ACEs = Adverse childhood experiences; RR = Relative Risk.

Female children were 92% more likely (RR = 1.92; 95% CI: 1.65–2.22) and 48% more likely (RR = 1.48; 95% CI: 1.32–1.65) to develop depression at ages 14–15 years and 16–17 years respectively, compared to males at the respective ages. Children with primary caregivers aged less than 27 years at childbirth had a 36% (RR: 1.36; 95% CI: 1.11–1.68) and 22% (RR = 1.22; 95% CI: 1.03–1.45) higher risk of depression at ages 12–13 and 14–15 years respectively, than their counterparts. Children from disadvantaged backgrounds were 35% more likely (RR = 1.35; 95% CI: 1.11–1.65) to develop depression at ages 12–13 years and had a 24% higher risk of depression at ages 16–17 years (RR = 1.24; 95% CI: 1.09–1.42), compared to those from advantaged backgrounds at respective ages. Children born to non-Australian born parents had 10% lower risk (RR = 0.90; 95% CI: 0.80–0.99) of reporting depression at ages 16–17 years compared to Australian parents.

### Association between parental history of ACEs, child’s ACEs exposure and adolescent anxiety at different ages

In the final adjusted interaction model, high threat- and deprivation-related ACEs, and parental ACEs were not significant at any ages. The three-way interaction was also not significant. However, in the adjusted model without interaction, children exposed to ≥ 2 deprivation-related ACEs had a 86% (RR = 1.86, 95% CI: 1.35–2.56), 51% (RR: 1.51; 95% CI: 1.14–2.01) higher risk of anxiety at ages 14–15 years and 57% (RR = 1.57; 95% CI: 1.24–1.97) higher risk at ages 16–17 years, compared to children exposed to 1 or less ACEs at respective ages (Table 4). Similarly, children exposed to ≥ 2 threat-related ACEs had a 50% (RR = 1.50; 95% CI: 1.10–2.03) higher risk of anxiety at ages 14–15 years.

**Table 4.** Modified Poisson regression model for the association between parental history of ACEs, child’s ACEs exposure (ages 3–16 years), and adolescent anxiety (ages 12–17 years)

	Anxiety at 12−13 years			Anxiety at 14−15 years			Anxiety at 16−17 years		
	Crude RR (95% CI)	Adjusted RR^a^ (95% CI)	Adjusted RR^a^ with interactions (95% CI)	Crude RR (95% CI)	Adjusted RR^a^ (95% CI)	Adjusted RR^a^ with interactions (95% CI)	Crude RR (95% CI)	Adjusted RR^a^ (95% CI)	Adjusted RR^a^ with interactions (95% CI)
**Study child’s sex**									
Male									
Female	0.90 (0.67−1.20)	0.89 (0.67−1.19)	0.89 (0.67−1.19)	2.08 (1.58−2.75)	2.16 (1.64−2.85)	2.17 (1.65−2.86)	1.39 (1.12−1.74)	1.41 (1.13−1.76)	1.40 (1.12−1.75)
**Age of the primary caregiver at birth of the study child**									
More than 27 years									
Less than or equal to 27 years	1.27 (0.90−1.79)	1.06 (0.74−1.53)	1.07 (0.74−1.53)	1.07 (0.77−1.5)	1.00 (0.71−1.41)	1.00 (0.71−1.40)	1.30 (0.99−1.71)	1.19 (0.90−1.57)	1.17 (0.88−1.55)
**Family socioeconomic position at Wave 1**									
Advantaged (Top 75%)									
Disadvantaged (Bottom 25%)	1.64 (1.20−2.24)	1.43 (1.03−1.98)	1.43 (1.03−1.99)	1.17 (0.85−1.60)	1.00 (0.72−1.38)	0.99 (0.72−1.37)	1.31 (1.01−1.70)	1.10 (0.84−1.45)	1.10 (0.84−1.45)
**Parents’ migrant status at Wave 1**									
Australian born									
Non-Australian born	0.94 (0.70−1.25)	0.94 (0.70−1.26)	0.94 (0.70−1.25)	1.14 (0.88−1.47)	1.11 (0.86−1.44)	1.12 (0.86−1.45)	1.08 (0.86−1.34)	1.06 (0.86−1.33)	1.06 (0.85−1.33)
**Parental ACEs (≥2)**									
No/Limited (0 to 1 ACE)									
Multiple (2 or more ACEs)	1.20 (0.88−1.63)	1.12 (0.82−1.52)	1.08 (0.73−1.60)	1.21 (0.92−1.59)	1.18 (0.90−1.55)	1.06 (0.74−1.52)	1.06 (0.84−1.33)	1.00 (0.8−1.26)	0.90 (0.66−1.25)
**Study child’s high threat related ACEs (≥2)**									
No									
Yes	1.31 (0.90−1.91)	1.04 (0.71−1.51)	1.01 (0.41−2.47)	1.56 (1.16−2.11)	1.50 (1.10−2.03)	1.14 (0.55−2.36)	1.45 (1.13−1.86)	1.29 (1.00−1.67)	1.42 (0.83−2.43)
**Study child’s high deprivation related ACEs (≥2)**									
No									
Yes	2.05 (1.52−2.77)	1.86 (1.35−2.56)	1.57 (0.82−3.03)	1.66 (1.26−2.17)	1.51 (1.14−2.01)	1.56 (0.91−2.67)	1.70 (1.36−2.12)	1.57 (1.24−1.97)	1.18 (0.72−1.92)
**Parental ACEs × High Threat ACEs × High Deprivation ACEs**									
Parental ACEs × High Threat ACEs	0.90 (0.25−3.32)	–	0.92 (0.25−3.36)	1.57 (0.60−4.14)	–	1.61 (0.61−4.25)	0.57 (0.24−1.36)	–	0.60 (0.25−1.42)
Parental ACEs × High Deprivation ACEs	1.26 (0.52−3.05)	–	1.29 (0.53−3.14)	1.00 (0.45−2.20)	–	1.01 (0.46−2.21)	1.29 (0.66−2.53)	–	1.28 (0.66−2.52)
High Threat ACEs × High Deprivation ACEs	1.27 (0.29−5.50)	–	1.33 (0.31−5.66)	0.80 (0.20−3.13)	–	0.76 (0.20−2.98)	0.80 (0.30−2.14)	–	0.78 (0.29−2.11)
Parental ACEs × High Threat ACEs × High Deprivation ACEs	1.21 (0.20−7.43)	–	1.16 (0.19−7.02)	1.38 (0.29−6.62)	–	1.54 (0.32−7.33)	1.60 (0.48−5.37)	–	1.59 (0.47−5.35)

a Adjusted for study child’s sex, age of the primary caregiver at birth of the study child, family socioeconomic position at Wave 1, Parents’ migrant status and Parental ACEs; ACEs = Adverse childhood experiences; RR = Relative Risk.

Compared to the males at respective ages, female children were 2.17 times more likely (RR = 2.17; 95% CI: 1.65–2.85) and had a 40% higher risk (RR = 1.40; 95% CI: 1.12–1.75) of anxiety at ages 14–15 years and 16–17 years respectively. Children from disadvantaged backgrounds were 43% more likely (RR = 1.43; 95% CI: 1.03–1.99) to develop anxiety at ages 12–13 years compared to those from advantaged backgrounds at respective ages.

### Sensitivity analysis

The results from the complete-case analyses and using continuous symptom scores were largely consistent with those from the imputed regressions. Detailed results are presented in Supplementary Tables S3-S6.

## Discussion

The study findings highlight the prevalence of depression and anxiety throughout adolescence, with depression rising from 11.2% at ages 12–13 years to 30.4% at 16–17 years and anxiety increasing from 4.5% to 9.4% over the same period. As hypothesized, children who were exposed to high deprivation-related ACEs consistently showed elevated rates of depression, with deprivation-related ACEs showing a stronger and more persistent association. Female adolescents faced a significantly higher risk of both depression and anxiety. Children from disadvantaged backgrounds and those born to younger caregivers (<27 years) also showed an elevated risk of depression. Deprivation-related ACEs maintained a strong link to depression across all stages of adolescence, while threat-related ACEs had a weaker and less consistent impact. The interaction of parental ACEs with threat- and deprivation-related ACEs did not show any significant results.

In our study, deprivation-related ACEs were consistently associated with the risk of depression throughout the adolescence, which may reflect disruptions in neurodevelopmental processes underlying emotion regulation, cognitive control, and attachment, consistent with psychiatric models of affective disorders (McLaughlin *et al.*, 2014; Sheridan and McLaughlin, 2014). One possibility is the specificity of intergenerational transmission mechanisms, where parental ACEs influence child depression through parental mental health (Johnson *et al.*, 2018), emotional dysregulation (Loechner *et al.*, 2020), or parenting styles (Romero-Acosta *et al.*, 2021), which are more strongly linked to affective disorders than to anxiety. In contrast, anxiety in adolescents may be more shaped by proximal environmental factors (e.g., school stress, peer relationships, or individual temperament) and acute threat exposure, which activate fear-related neural circuits, including the amygdala and salience network (Degnan *et al.*, 2010). This pattern may also suggest differential vulnerability pathways, consistent with theories that deprivation-threat model of ACEs, where deprivation impacts cognitive and emotional development leading to depression, whereas threat exposure more strongly influences anxiety and stress reactivity (Miller *et al.*, 2018; Uddin *et al.*, 2024).

The stronger and more persistent association between deprivation-related ACEs and depression may reflect the distinct developmental consequences of early-life deprivation. Unlike threat, which typically involves acute danger and activates fear-related systems, deprivation often entails chronic under-enrichment during sensitive developmental periods, disrupting neurocircuitry supporting executive function and emotion regulation, and increasing vulnerability to depressive disorders over time (McLaughlin *et al.*, 2014; Sheridan and McLaughlin, 2014). Early childhood represents a sensitive period for brain development, and deprivation during this period may produce more enduring psychiatric consequences than later exposures (Mackes *et al.*, 2020).

Parental ACEs were associated with child depression, pointing to a potential intergenerational transmission of psychiatric vulnerability. Parents with a history of ACEs may exhibit altered parenting behaviours, emotional availability, and attachment disruptions, making it more challenging to create a stable and supportive environment for their children (Cooke *et al.*, 2019; Rowell and Neal-Barnett, 2022). Addressing ACEs within Australia’s public health system may benefit from family-focused, trauma-informed approaches targeting both parents and children, with the goal of breaking the cycle of trauma and its psychological impact across generations (Haynes *et al.*, 2020). Such interventions should build on parents’ existing strengths and be tailored to their socio-cultural context to foster belonging, empowerment, and safety, thereby mitigating the effects of disconnection and providing a foundation for resilience (Woods-Jaeger *et al.*, 2018). Strength-based strategies could include raising awareness of ACEs, fostering nurturing parent-child relationships, and providing accessible, culturally responsive trauma-informed parenting programs (Gupta *et al.*, 2021).

Screening for parental ACEs can help identify children at heightened risk for psychiatric disorder, enabling early interventions to prevent downstream consequences such as academic struggles, mental illness, and substance use (Schickedanz *et al.*, 2018). Future directions could include antenatal ACE screening, teacher and counsellor training for early identification of at-risk children, and enhanced community mental health outreach. Pilot programs in the USA and UK demonstrate the feasibility of integrating ACE screening into routine (mental) healthcare, providing models for trauma-informed interventions that could be carefully adapted to the Australian context (Flanagan *et al.*, 2018; Mortimore *et al.*, 2021).

Deprivation, such as economic hardship, housing instability, and parental neglect, remained a consistent predictor for depression in children, highlighting the need for structural policies and programs to address socioeconomic disadvantage. Given ongoing inequalities in wealth distribution and access to education, mental healthcare and social support, policies aimed at reducing childhood poverty and improving access to essential services like healthcare, education, and social support may play a vital role in mitigating these risks. In socioeconomically disadvantaged regions, such interventions would substantially reduce the incidence of depression later in life.

However, Australia currently lacks trauma-informed policies and interventions specifically designed for vulnerable children and families. This gap is partly attributable to mental health resources in rural and regional areas, and to implementation barriers such as concerns over privacy, acceptability, and workforce capacity (Tran *et al.*, 2022). To be effective, interventions must move beyond individual-level support (e.g., trauma-focused care) and address both proximal environments (e.g., responsive parenting) and distal contexts (e.g., safe schools and communities) (Bhutta *et al.*, 2023). Developmentally tailored parenting programs could equip caregivers with essential skills to buffer mental health risk during critical periods, consistent with evidence from developmental psychiatry and the deprivation-threat ACEs framework.

### Strengths and limitations

The strength of this study is that it utilised national-level longitudinal data to examine the relationship between parental ACEs, threat- and deprivation-related ACEs, and depression and anxiety at different ages.

However, these findings should be interpreted with caution given some potential limitations. First, the LSAC did not collect standard variables on childhood adversities, such as physical or sexual abuse, but instead included retrospectively collected measures of child maltreatment. Some ACE-related variables were proxies for child maltreatment, potentially subjecting to reporting and recall bias. Second, measures of parental ACEs were also assessed retrospectively, which may be influenced by recall bias. Third, the LSAC sample underrepresents children from low socioeconomic backgrounds, Aboriginal and Torres Strait Islander families, and immigrant families, potentially leading to an underestimation of the association between ACEs and mental disorders in these specific subgroups. And last, residual confounding cannot be ruled out despite statistical adjustments. Addressing these limitations in future research would enhance the generalizability and robustness of findings in this area.

## Conclusion

This study highlights the profound impact of early-life deprivations rooted in financial hardship and familial adversities on the adolescent mental health outcomes in Australia. Additionally, children born to parents with a history of multiple ACEs were found to have a heightened risk of depression during adolescence. There is a need for targeted interventions that address mental health needs of children exposed to economic hardship, housing instability, and parental neglect. Early identification of vulnerable children, combined with multi-level interventions, spanning individual, family and policy-driven approaches, addressing the root causes of deprivation-related ACEs could significantly reduce the mental health burden on future generations.

## Supporting information

10.1017/S2045796025100255.sm001 Giri et al. supplementary material Giri et al. supplementary material

## Data Availability

The data used in this study are not publicly available. Access to the Longitudinal Study of Australian Children (LSAC) dataset can be requested through the Australian Data Archive Dataverse, managed by the Australian Government Department of Social Services, via https://dataverse.ada.edu.au/.

## List of abbreviations

ACEs: Adverse Childhood Experiences
CAS: Children’s Anxiety Scale
CI: Confidence Interval
DMAP: Dimensional Model of Psychopathology
LSAC: Longitudinal Study of Children
RR: Relative Risk
SEP: Socio-Economic Position
SMFQ: Short Mood and Feelings Questionnaire

## Supplementary material

The supplementary material for this article can be found at https://doi.org/10.1017/S2045796025100255.
