# Supplementary material for: The effects of parental adverse childhood experiences (ACEs) and childhood threat and deprivation on adolescent depression and anxiety: an analysis of the longitudinal study of Australian children
Source: Epidemiol Psychiatr Sci. 2025 Oct 6;34:e49. doi: 10.1017/S2045796025100255 (PMC12555079; doi:10.1017/S2045796025100255)
Supplement: Giri et al. supplementary material [file S2045796025100255sup001.docx]

**Table S1. Definitions of the children’s ACE variables**

| **Variables** | **Definition/Operationalisation** |
| --- | --- |
| **Threat-related ACEs** | |
| Interparental Violence | Based on the question: “How often do you have arguments with your partner that end up with people pushing, hitting, kicking, or shoving?” with a 4-point response (Never; Rarely; Sometimes; Often; Always) at Waves 1-7. Grouped as ‘0’ = No, for Never, and ‘1’ = Yes, for ‘Rarely’, ‘Sometimes’, ‘Often’, and ‘Always’. |
| Harsh Parenting | Parents reported frequency of hostile behaviours (hostility, aggression, irritability) toward their child over the past 4 weeks, on a scale from "never" to "always." Top 5% within each wave (1-7) dichotomised as harsh parenting. Binary: 0 = ‘No’, if neither parent reported harsh parenting, 1 = ‘Yes’, if harsh parenting by either parent. |
| Unsafe neighbourhood | Derived from the question: “How strongly do you agree or disagree with these statements about your neighbourhood? This is a safe neighbourhood” asked to parent 1 from Waves 1-6 (answered on a scale of 1 Strongly agree; 2 Agree; 3 Disagree; 4 Strongly disagree), and from “How safe do you feel walking alone in the area near your home during the daytime?” and “How safe do you feel walking alone in the area near your home after dark?” from the study child at Wave 7 or 16-17 years (answered in the scale of 1 Completely safe; 2 Very safe; 3 Fairly safe; 4 Neither safe nor unsafe; 5 Fairly unsafe; 6 Very unsafe; 7 Not at all safe). For parents, who answered 3 ‘Disagree’ or 4 ‘Strongly disagree’ and for study children who answered, ‘Fairly unsafe’, ‘Very unsafe’, or ‘Not at all safe’ were coded as 1 for ‘Yes’ and the rest of the responses as 0 ‘No’ for unsafe neighbourhood. |
| Bullying Victimization | Derived from reports by parent 1 and teacher on whether the child was “picked on or bullied by other children.” Binary indicator: 0 = ‘No’, if no bullying reported, 1 = ‘Yes’, if bullying reported by parent 1 and/or teacher at each wave. |
| **Deprivation-related ACEs** | |
| Financial Hardship | Assessed in each wave through questions on household economic difficulties (e.g., unpaid bills, skipped meals, pawning items, or welfare reliance). Scored (1 = Yes, 0 = No); scores summed for a hardship score. Binary: 0 = ‘No’, for 0 to 1 hardship, 1 = ‘Yes’, for two or more financial hardships in any wave. |
| Parental Legal Problems | Derived from the question: “In the last year, have any of the following happened to you? You had problems with the police and a court appearance,” with Yes/No responses. Binary indicator: 0 = ‘No’, 1 = ‘Yes’, for legal problems in any wave. |
| Parental Psychological Distress | Measured using the six-item Kessler Psychological Distress Scale (K-6). Each parent reported their distress on a five-point scale. Responses summed for a total score. A cut-off score of 13+ indicates distress. Binary: 0 = ‘No’, for neither parent distressed, 1 = ‘Yes’, for parent 1 and/or parent 2 distressed at each wave. |
| Household Substance Abuse | Asked with parent 1: “In the last year, have any of the following happened to you? Someone in your household had an alcohol or drug problem.” Binary indicator: 0 = ‘No’, for no substance abuse, 1 = ‘Yes’, for the presence of substance abuse in the household at each wave. |
| Parental Separation/Divorce | Measured by asking if the parents had any separation due to relationship or marital difficulties. Binary indicator: 0 = ‘No’, for no separation/divorce, 1 = ‘Yes’, if separation/divorce occurred at each wave. Single parent was coded as ‘0’. |
| Death of a Family Member | Parent 1 asked: “In the last year, have any of the following happened to you? Your parent, partner, or child died.” at each wave. Binary indicator: 0 = ‘No’, 1 = ‘Yes’ |

**Table S2. Definitions of the parent’s ACE variables**

| **Variables** | **Definition/Operationalisation** |
| --- | --- |
| Authoritarian Upbringing | Based on the question “During your childhood, did you experience any of the following? Had an authoritarian, or regimented upbringing?” asked to both parents at Wave 7. Responses were dichotomised: 0 = No, 1 = Yes. |
| Live in Foster Family/Welfare Home/Institution | Based on the question “During your childhood, did you experience any of the following? Live in foster family/welfare home/institution?” asked to both parents at Wave 7. Dichotomised as 0 = No and 1 = Yes. |
| Financial Hardship | Derived from the question “During your childhood, did you experience any of the following? Family was poor and struggled to make ends meet” asked to both parents at Wave 7. Binary coding: 0 = No; 1 = Yes. |
| Interparental Violence | Captures exposure to conflict between parents. Items include: During your childhood, did you experience any of the following?   - “There were frequent arguments between parents” (Wave 7) - “Father physically abused mother” (Wave 7) - “Mother physically abused father” (Wave 7) - “Father verbally abused mother” (Wave 7) - “Mother verbally abused father” (Wave 7) - “Conflict when growing up” (Wave 2)   If any item is endorsed (0 = No; 1 = Yes), the indicator is coded as 1. |
| Parental Mental Illness | Based on the questions “During your childhood, did you experience any of the following? Father had mental illness” and “During your childhood, did you experience any of the following? Mother had mental illness” asked at Wave 2. Dichotomised as 0 = No (if neither parent reported mental illness) and 1 = Yes (if either parent did). |
| Parental Alcohol/Drug Problem | Derived from the questions “During your childhood, did you experience any of the following? Father had drinking/drug problem” and “During your childhood, did you experience any of the following? Mother had drug/alcohol problem” asked at Wave 2. Binary indicator: 0 = No; 1 = Yes if either parent reported substance-related problems. |
| Neglect | Combines two items: During your childhood, did you experience any of the following?  “Frequently left alone to look after yourself” (Wave 7)  “There was often not enough food in the house” (Wave 7)  Dichotomised: 0 = No; 1 = Yes if either parent reported yes. |
| Physically Abused | Based on the question “During your childhood, did you experience any of the following? Received frequent beatings or too much punishment” asked to both parents at Wave 7. Responses were dichotomised (0 = No; 1 = Yes). |
| Sexually Abused | Includes three items:  “During your childhood, did you experience any of the following? Sexually abused by family member in household”  “During your childhood, did you experience any of the following? Sexually abused by family member not in household”  “When you were a teenager, before you were 18 years of age did any of the following occur? Were raped or sexually assaulted” All asked at Wave 7. Dichotomised: 0 = No; 1 = Yes if any item is endorsed. |
| Verbally Abused | Based on the question “During your childhood, did you experience any of the following? Verbally abused/ridiculed/humiliated by a parent” asked to both parents at Wave 7. Binary coding: 0 = No; 1 = Yes. |

**Table S3: Modified Poisson regression model for the association between parental history of ACEs, child’s ACEs exposure (Ages 3-16 years), and adolescent depression (Ages 12-17 years) using complete-case analysis**

|  | **Depression at 12 to 13 years** | | | **Depression at 14 to 15 years** | | | **Depression at 16 to 17 years** | | |
| --- | --- | --- | --- | --- | --- | --- | --- | --- | --- |
|  | **Crude RR (95% CI)** | **Adjusted RR* (95% CI)** | **Adjusted RR* with interactions (95% CI)** | **Crude RR (95% CI)** | **Adjusted RR* (95% CI)** | **Adjusted RR* with interactions (95% CI)** | **Crude RR (95% CI)** | **Adjusted RR* (95% CI)** | **Adjusted RR* with interactions (95% CI)** |
| **Study child's sex** |  |  |  |  |  |  |  |  |  |
| Male | Ref. | Ref. | Ref. | Ref. | Ref. | Ref. | Ref. | Ref. | Ref. |
| Female | 1.24 (1.02–1.5) | 1.21 (0.99–1.48) | 1.22 (0.99–1.49) | 1.93 (1.65–2.27) | 1.95 (1.65–2.3) | 1.94 (1.65–2.29) | 1.49 (1.31–1.68) | 1.45 (1.28–1.65) | 1.45 (1.28–1.65) |
| **Age of the primary caregiver at birth of the study child** |  |  |  |  |  |  |  |  |  |
| More than 27 years | Ref. | Ref. | Ref. | Ref. | Ref. | Ref. | Ref. | Ref. | Ref. |
| Less than or equal to 27 years | 1.37 (1.14–1.64) | 1.32 (1.03–1.68) | 1.31 (1.03–1.67) | 1.31 (1.09–1.57) | 1.2 (0.99–1.46) | 1.2 (0.99–1.46) | 1.18 (1.01–1.38) | 1.08 (0.92–1.27) | 1.08 (0.92–1.27) |
| **Family socioeconomic position at Wave 1** |  |  |  |  |  |  |  |  |  |
| Advantaged (Top 75%) | Ref. | Ref. | Ref. | Ref. | Ref. | Ref. | Ref. | Ref. | Ref. |
| Disadvantaged (Bottom 25%) | 1.63 (1.33–2) | 1.32 (1.04–1.67) | 1.32 (1.04–1.66) | 1.26 (1.06–1.51) | 1.12 (0.93–1.35) | 1.12 (0.93–1.35) | 1.38 (1.21–1.59) | 1.26 (1.09–1.46) | 1.26 (1.09–1.45) |
| **Parent's migrant status at Wave 1** |  |  |  |  |  |  |  |  |  |
| Australian born | Ref. | Ref. | Ref. | Ref. | Ref. | Ref. | Ref. | Ref. | Ref. |
| Non-Australian born | 0.96 (0.79–1.17) | 1 (0.82–1.22) | 0.99 (0.81–1.22) | 0.99 (0.85–1.16) | 1.01 (0.87–1.18) | 1.01 (0.87–1.18) | 0.87 (0.76–0.98) | 0.89 (0.79–1.01) | 0.89 (0.79–1.01) |
| **Parental ACEs (≥2)** |  |  |  |  |  |  |  |  |  |
| No/Limited (0 to 1 ACE) | Ref. | Ref. | Ref. | Ref. | Ref. | Ref. | Ref. | Ref. | Ref. |
| Multiple (2 or more ACEs) | 1.41 (1.15–1.74) | 1.29 (1.04–1.6) | 1.46 (1.1–1.93) | 1.11 (0.94–1.3) | 1.07 (0.91–1.26) | 1.01 (0.82–1.25) | 1.11 (0.98–1.27) | 1.1 (0.97–1.26) | 1.17 (0.98–1.39) |
| **Study child's high threat related ACEs (≥2)** |  |  |  |  |  |  |  |  |  |
| No | Ref. | Ref. | Ref. | Ref. | Ref. | Ref. | Ref. | Ref. | Ref. |
| Yes | 1.54 (1.22–1.96) | 1.24 (0.96–1.62) | 1.85 (1.1–3.14) | 1.29 (1.07–1.55) | 1.1 (0.9–1.33) | 0.91 (0.57–1.45) | 1.19 (1.02–1.37) | 1.1 (0.95–1.28) | 1.35 (0.99–1.85) |
| **Study child's high deprivation related ACEs (≥2)** |  |  |  |  |  |  |  |  |  |
| No | Ref. | Ref. | Ref. | Ref. | Ref. | Ref. | Ref. | Ref. | Ref. |
| Yes | 2.09 (1.72–2.54) | 1.79 (1.42–2.25) | 2.56 (1.68–3.91) | 1.64 (1.4–1.92) | 1.61 (1.36–1.91) | 1.44 (1.06–1.97) | 1.34 (1.18–1.52) | 1.23 (1.07–1.41) | 1.45 (1.12–1.87) |
| **Parental ACEs X High Threat ACEs X High Deprivation ACEs** |  |  |  |  |  |  |  |  |  |
| Parental ACEs X High Threat ACEs | 1.04 (0.5–2.17) | - | 1.06 (0.51–2.21) | 1.32 (0.7–2.49) | - | 1.32 (0.71–2.48) | 0.87 (0.55–1.37) | - | 0.92 (0.59–1.45) |
| Parental ACEs X High Deprivation ACEs | 0.97 (0.54–1.74) | - | 0.99 (0.55–1.79) | 1.08 (0.69–1.7) | - | 1.18 (0.76–1.83) | 0.95 (0.66–1.37) | - | 0.97 (0.67–1.39) |
| High Threat ACEs X High Deprivation ACEs | 0.5 (0.18–1.37) | - | 0.54 (0.19–1.48) | 1.42 (0.69–2.94) | - | 1.47 (0.71–3.05) | 0.69 (0.38–1.25) | - | 0.68 (0.38–1.22) |
| Parental ACEs X High Threat ACEs X High Deprivation ACEs | 3.61 (1.12–11.63) | - | 3.3 (1.02–10.76) | 0.64 (0.27–1.53) | - | 0.63 (0.26–1.5) | 2.25 (1.12–4.51) | - | 2.1 (1.06–4.19) |

*Adjusted for study child’s sex, age of the primary caregiver at birth of the study child, family socioeconomic position at Wave 1, Parent’s migrant status and Parental ACEs; ACEs= Adverse childhood experiences; RR=Relative Risk

**Table S4: Modified Poisson regression model for the association between parental history of ACEs, child’s ACEs exposure (Ages 3-16 years), and adolescent anxiety (Ages 12-17 years) using complete-case analysis**

|  | **Anxiety at 12 to 13 years** | | | **Anxiety at 14 to 15 years** | | | **Anxiety at 16 to 17 years** | | |
| --- | --- | --- | --- | --- | --- | --- | --- | --- | --- |
|  | **Crude RR (95% CI)** | **Adjusted RR* (95% CI)** | **Adjusted RR* with interactions (95% CI)** | **Crude RR (95% CI)** | **Adjusted RR* (95% CI)** | **Adjusted RR* with interactions (95% CI)** | **Crude RR (95% CI)** | **Adjusted RR* (95% CI)** | **Adjusted RR* with interactions (95% CI)** |
| **Study child's sex** |  |  |  |  |  |  |  |  |  |
| Male | Ref. | Ref. | Ref. | Ref. | Ref. | Ref. | Ref. | Ref. | Ref. |
| Female | 0.94 (0.69–1.29) | 0.84 (0.61–1.17) | 0.85 (0.61–1.17) | 2.15 (1.59–2.9) | 2.2 (1.61–3.01) | 2.2 (1.61–3.01) | 1.4 (1.1–1.8) | 1.39 (1.08–1.79) | 1.39 (1.08–1.78) |
| **Age of the primary caregiver at birth of the study child** |  |  |  |  |  |  |  |  |  |
| More than 27 years | Ref. | Ref. | Ref. | Ref. | Ref. | Ref. | Ref. | Ref. | Ref. |
| Less than or equal to 27 years | 1.18 (0.82–1.7) | 1.13 (0.75–1.7) | 1.14 (0.76–1.71) | 1.14 (0.79–1.65) | 1.01 (0.68–1.51) | 1.01 (0.68–1.51) | 1.3 (0.96–1.77) | 1.16 (0.85–1.6) | 1.15 (0.84–1.58) |
| **Family socioeconomic position at Wave 1** |  |  |  |  |  |  |  |  |  |
| Advantaged (Top 75%) | Ref. | Ref. | Ref. | Ref. | Ref. | Ref. | Ref. | Ref. | Ref. |
| Disadvantaged (Bottom 25%) | 1.55 (1.11–2.15) | 1.28 (0.86–1.89) | 1.27 (0.85–1.89) | 1.13 (0.8–1.58) | 1.02 (0.7–1.48) | 1.02 (0.7–1.47) | 1.3 (0.98–1.73) | 1.08 (0.8–1.47) | 1.08 (0.79–1.46) |
| **Parent's migrant status at Wave 1** |  |  |  |  |  |  |  |  |  |
| Australian born | Ref. | Ref. | Ref. | Ref. | Ref. | Ref. | Ref. | Ref. | Ref. |
| Non-Australian born | 0.95 (0.7–1.3) | 0.82 (0.58–1.15) | 0.82 (0.58–1.16) | 1.2 (0.91–1.6) | 1.33 (0.99–1.79) | 1.34 (1–1.8) | 1.03 (0.81–1.32) | 1.05 (0.81–1.34) | 1.04 (0.81–1.34) |
| **Parental ACEs (≥2)** |  |  |  |  |  |  |  |  |  |
| No/Limited (0 to 1 ACE) | Ref. | Ref. | Ref. | Ref. | Ref. | Ref. | Ref. | Ref. | Ref. |
| Multiple (2 or more ACEs) | 1.23 (0.88–1.72) | 1.06 (0.76–1.49) | 1.05 (0.68–1.6) | 1.19 (0.88–1.61) | 1.21 (0.9–1.65) | 0.97 (0.65–1.45) | 1.1 (0.85–1.42) | 1.07 (0.83–1.39) | 0.88 (0.61–1.27) |
| **Study child's high threat related ACEs (≥2)** |  |  |  |  |  |  |  |  |  |
| No | Ref. | Ref. | Ref. | Ref. | Ref. | Ref. | Ref. | Ref. | Ref. |
| Yes | 1.34 (0.9–2.01) | 1.16 (0.75–1.8) | 1.15 (0.41–3.21) | 1.56 (1.12–2.18) | 1.45 (1.02–2.06) | 1.02 (0.45–2.35) | 1.52 (1.15–2) | 1.38 (1.03–1.84) | 1.45 (0.8–2.66) |
| **Study child's high deprivation related ACEs (≥2)** |  |  |  |  |  |  |  |  |  |
| No | Ref. | Ref. | Ref. | Ref. | Ref. | Ref. | Ref. | Ref. | Ref. |
| Yes | 1.99 (1.44–2.76) | 1.89 (1.31–2.72) | 1.64 (0.78–3.46) | 1.69 (1.26–2.27) | 1.52 (1.09–2.12) | 1.41 (0.77–2.59) | 1.67 (1.31–2.14) | 1.63 (1.25–2.12) | 1.26 (0.73–2.17) |
| **Parental ACEs X High Threat ACEs X High Deprivation ACEs** |  |  |  |  |  |  |  |  |  |
| Parental ACEs X High Threat ACEs | 0.73 (0.17–3.18) | - | 0.74 (0.17–3.26) | 1.83 (0.61–5.54) | - | 1.89 (0.63–5.71) | 0.71 (0.28–1.84) | - | 0.75 (0.29–1.94) |
| Parental ACEs X High Deprivation ACEs | 1.14 (0.42–3.07) | - | 1.13 (0.42–3.06) | 1.15 (0.48–2.75) | - | 1.17 (0.49–2.78) | 1.3 (0.61–2.77) | - | 1.29 (0.61–2.73) |
| High Threat ACEs X High Deprivation ACEs | 1.09 (0.22–5.55) | - | 1.12 (0.22–5.62) | 0.69 (0.13–3.78) | - | 0.68 (0.12–3.79) | 0.6 (0.19–1.86) | - | 0.59 (0.19–1.82) |
| Parental ACEs X High Threat ACEs X High Deprivation ACEs | 1.81 (0.24–13.55) | - | 1.74 (0.23–13.08) | 1 (0.15–6.62) | - | 1.11 (0.17–7.5) | 1.59 (0.41–6.22) | - | 1.58 (0.4–6.17) |

*Adjusted for study child’s sex, age of the primary caregiver at birth of the study child, family socioeconomic position at Wave 1, Parent’s migrant status and Parental ACEs; ACEs= Adverse childhood experiences; RR=Relative Risk

**Table S5: Modified Poisson regression model for the association between parental history of ACEs, child’s ACEs exposure (Ages 3-16 years), and adolescent anxiety (Ages 12-17 years) using continuous symptom scores of depression**

|  | **Depression at 12 to 13 years** | **Depression at 14 to 15 years** | **Depression at 16 to 17 years** |
| --- | --- | --- | --- |
|  | **Adjusted RR* (95% CI)** | **Adjusted RR* (95% CI)** | **Adjusted RR* (95% CI)** |
| **Study child's sex** |  |  |  |
| Male | Ref. | Ref. | Ref. |
| Female | 1.13 (1.04–1.22) | 1.54 (1.42–1.67) | 1.35 (1.25–1.45) |
| **Age of the primary caregiver at birth of the study child** |  |  |  |
| More than 27 years | Ref. | Ref. | Ref. |
| Less than or equal to 27 years | 1.13 (1.02–1.25) | 1.13 (1.02–1.26) | 1.04 (0.94–1.15) |
| **Family socioeconomic position at Wave 1** |  |  |  |
| Advantaged (Top 75%) | Ref. | Ref. | Ref. |
| Disadvantaged (Bottom 25%) | 1.16 (1.05–1.27) | 1.13 (1.03–1.25) | 1.16 (1.06–1.26) |
| **Parent's migrant status at Wave 1** |  |  |  |
| Australian born | Ref. | Ref. | Ref. |
| Non-Australian born | 0.98 (0.9–1.07) | 0.99 (0.91–1.07) | 0.95 (0.89–1.03) |
| **Parental ACEs (≥2)** |  |  |  |
| No/Limited (0 to 1 ACE) | Ref. | Ref. | Ref. |
| Multiple (2 or more ACEs) | 1.11 (1.02–1.21) | 1.09 (1–1.18) | 1.12 (1.04–1.21) |
| **Study child's high threat related ACEs (≥2)** |  |  |  |
| No | Ref. | Ref. | Ref. |
| Yes | 1.2 (1.08–1.35) | 1.18 (1.07–1.3) | 1.07 (0.98–1.17) |
| **Study child's high deprivation related ACEs (≥2)** |  |  |  |
| No | Ref. | Ref. | Ref. |
| Yes | 1.33 (1.21–1.47) | 1.3 (1.19–1.42) | 1.15 (1.07–1.25) |

*Adjusted for study child’s sex, age of the primary caregiver at birth of the study child, family socioeconomic position at Wave 1, Parent’s migrant status and Parental ACEs; ACEs= Adverse childhood experiences; RR=Relative Risk

**Tables S6: Modified Poisson regression model for the association between parental history of ACEs, child’s ACEs exposure (Ages 3-16 years), and adolescent anxiety (Ages 12-17 years) using continuous symptom scores of anxiety**

|  | **Anxiety at 12 to 13 years** | **Anxiety at 14 to 15 years** | **Anxiety at 16 to 17 years** |
| --- | --- | --- | --- |
|  | **Adjusted RR* (95% CI)** | **Adjusted RR* (95% CI)** | **Adjusted RR* (95% CI)** |
| **Study child's sex** |  |  |  |
| Male | Ref. | Ref. | Ref. |
| Female | 1.26 (1.2–1.31) | 1.64 (1.55–1.73) | 1.65 (1.56–1.75) |
| **Age of the primary caregiver at birth of the study child** |  |  |  |
| More than 27 years | Ref. | Ref. | Ref. |
| Less than or equal to 27 years | 0.97 (0.91–1.03) | 0.96 (0.89–1.03) | 1.01 (0.93–1.09) |
| **Family socioeconomic position at Wave 1** |  |  |  |
| Advantaged (Top 75%) | Ref. | Ref. | Ref. |
| Disadvantaged (Bottom 25%) | 1.02 (0.96–1.09) | 1.02 (0.96–1.1) | 1.05 (0.98–1.14) |
| **Parent's migrant status at Wave 1** |  |  |  |
| Australian born | Ref. | Ref. | Ref. |
| Non-Australian born | 1 (0.95–1.05) | 1.05 (0.99–1.1) | 1.06 (1–1.12) |
| **Parental ACEs (≥2)** |  |  |  |
| No/Limited (0 to 1 ACE) | Ref. | Ref. | Ref. |
| Multiple (2 or more ACEs) | 1.05 (1–1.1) | 1.09 (1.03–1.15) | 1.06 (1–1.13) |
| **Study child's high threat related ACEs (≥2)** |  |  |  |
| No | Ref. | Ref. | Ref. |
| Yes | 1.06 (0.99–1.13) | 1.09 (1.02–1.17) | 1.11 (1.03–1.19) |
| **Study child's high deprivation related ACEs (≥2)** |  |  |  |
| No | Ref. | Ref. | Ref. |
| Yes | 1.17 (1.1–1.24) | 1.11 (1.05–1.19) | 1.13 (1.06–1.21) |

*Adjusted for study child’s sex, age of the primary caregiver at birth of the study child, family socioeconomic position at Wave 1, Parent’s migrant status and Parental ACEs; ACEs= Adverse childhood experiences; RR=Relative Risk
